# Safety and Efficacy of Concomitant Administration of Nanoliposomal Irinotecan + 5‐Fluorouracil/*Levo*‐Leucovorin for Pancreatic Cancer

**DOI:** 10.1002/cnr2.70485

**Published:** 2026-02-05

**Authors:** Akane Wakabayashi, Rei Tanaka, Yurie Fukumuro, Keita Mori, Junya Sato, Hiroshi Ishikawa, Michihiro Shino, Takeshi Kawakami

**Affiliations:** ^1^ Department of Pharmacy Shizuoka Cancer Center Sunto‐gun Shizuoka Japan; ^2^ Department of Pharmacy Shonan University of Medical Sciences Yokohama Kanagawa Japan; ^3^ Department of Nursing Shizuoka Cancer Center Sunto‐gun Shizuoka Japan; ^4^ Clinical Research Center Shizuoka Cancer Center Sunto‐gun Shizuoka Japan; ^5^ Division of Gastrointestinal Oncology Shizuoka Cancer Center Sunto‐gun Shizuoka Japan

**Keywords:** adverse events, concomitant administration, drug compatibility, nanoliposomal irinotecan, pancreas cancer

## Abstract

**Background:**

Nanoliposomal irinotecan (nal‐IRI) plus 5‐fluorouracil (5‐FU)/*levo*‐leucovorin (*Levo*‐LV) therapy is recommended as the standard of care for unresectable locally advanced (UR‐LA) and metastatic pancreatic cancer after failure of gemcitabine‐containing regimens. Although the concomitant administration of nal‐IRI and *Levo*‐LV benefits from a reduced hospital stay, nal‐IRI is usually administered after *Levo*‐LV owing to insufficient data on compatibility reactions. This study aimed to compare the safety and efficacy of the sequential and concomitant administration of nal‐IRI and *Levo*‐LV.

**Methods:**

Data of patients with UR‐LA or metastatic pancreatic cancer who received nal‐IRI plus 5‐FU/Levo‐LV between 2020 and 2023 at Shizuoka Cancer Center were retrospectively collected. Patients were classified into the sequential administration group (Group S) and concomitant administration group (Group C) to compare adverse events, infusion time, and survival. Univariate and multivariate analyses were performed to identify independent prognostic factors in each group.

**Results:**

A total of 94 patients were included (44 in Group S and 50 in Group C). There was no significant difference in the incidence of Grade 3 or higher adverse events between the two groups. The median total infusion times for nal‐IRI plus 5‐FU/Levo‐LV in Groups S and C were 271 and 149 min, respectively (*p* < 0.001). Overall survival estimates were 5.6 months (95% confidence interval [CI] 3.78–8.57) in Group S and 8.4 months (95% CI 6.77–10.3) in Group C (unstratified HR 0.65, 95% CI 0.42–1.02; *p* = 0.058). In the multivariate analysis for PFS and OS, the method of administration was not identified as an independent prognostic factor. Concomitant administration of *Levo*‐LV with nal‐IRI may not increase adverse events or impact efficacy while reducing infusion time.

**Conclusion:**

Concomitant administration of *Levo*‐LV with nal‐IRI may not increase adverse events or impact efficacy compared to sequential administration.

## 1 Introduction

Pancreatic cancer is associated with a poor prognosis. By 2022, 510 566 new cases and 467 005 deaths were reported worldwide, ranking 12th and 6th in terms of incidence and deaths [1], respectively. The number of deaths in Japan was 39 468, ranking 4th in terms of deaths by site in 2022, and this number has been increasing in recent years [2, 3].

Nanoliposomal irinotecan (nal‐IRI) is a topoisomerase I inhibitor consisting of irinotecan encapsulated in liposomal particles [4, 5]. Nal‐IRI has a longer plasma circulation time than those of non‐liposomal formulations, increased tumor accumulation due to increased vascular permeability and retention, and enhanced anti‐tumor activity due to a longer exposure period, particularly for the SN‐38 formulation [4, 5]. In the NAPOLI‐1 trial, the combination therapy of nal‐IRI with 5‐fluorouracil (5‐FU) and leucovorin (LV) prolonged median overall survival (OS) significantly in patients with pancreatic cancer with distant metastases after the failure of gemcitabine (GEM)‐based chemotherapy (6.2 vs. 4.2 months; unstratified hazard ratio [HR] 0.75; 95% CI 0.57–0.99; *p* < 0.001) [6]. In a Japanese randomized phase II trial, nal‐IRI in combination with 5‐FU/levo‐leucovorin (*Levo*‐LV) prolonged median progression‐free survival (PFS) significantly compared with that for 5‐FU/LV alone (2.7 vs. 1.5 months; HR 0.60; 95% CI 0.37–0.98; *p* = 0.039) [7]. The combination of nal‐IRI with 5‐FU/LV is recommended as a standard treatment for patients with metastatic pancreatic cancer after the failure of a GEM‐based regimen worldwide [8, 9, 10, 11].

**FIGURE 1:**
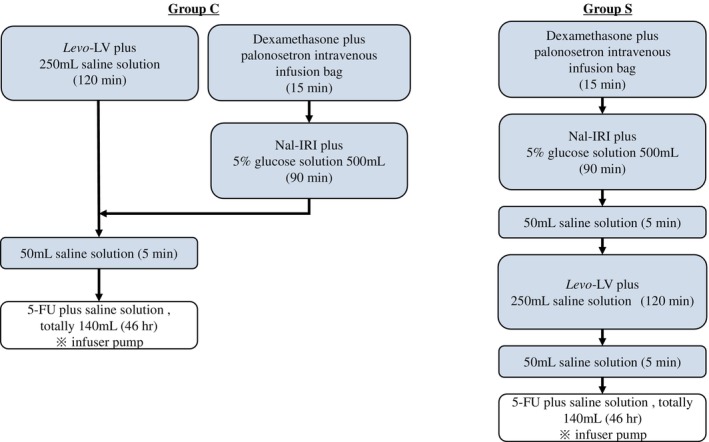
Method of administration. In Group C, nal‐IRI (70 mg/m^2^ as IRI) and *Levo*‐LV (200 mg/m^2^) were infused simultaneously over 90 and 120 min, respectively, followed by 5‐FU (2400 mg/m^2^) over 46 h (left figure). In Group S, nal‐IRI (70 mg/m^2^ as IRI) was infused over 90 min, followed by *Levo*‐LV (200 mg/m^2^) over 120 min, and then 5‐FU (2400 mg/m^2^) over 46 h (right figure). 5‐FU, 5‐fluorouracil; *Levo*‐LV, *Levo*‐leucovorin; Nal‐IRI, nanoliposomal irinotecan.

Although nal‐IRI and *Levo*‐LV are administered sequentially, there are some concerns regarding this approach. First, it takes approximately 4 h from the start of the regimen to switch to continuous 5‐FU infusion. Second, this method increases patient burden owing to a longer hospital stay. To address this issue, our hospital concomitantly administers nal‐IRI and *Levo*‐LV. In particular, nal‐IRI is administered from a side branch of *Levo*‐LV (Figure 1) based on safety data for the concomitant administration of IRI, which has the same mechanism of action as nal‐IRI and *Levo*‐LV. Parallel administration of nal‐IRI and *Levo*‐LV is clinically safe and not inferior in efficacy [12]; however, only a few reports have documented such cases. This study aimed to compare the safety and efficacy of combined and sequential administration of nal‐IRI and *Levo*‐LV at our institution.

## 2 Methods

### 2.1 Patients and Methods

**FIGURE 2:**
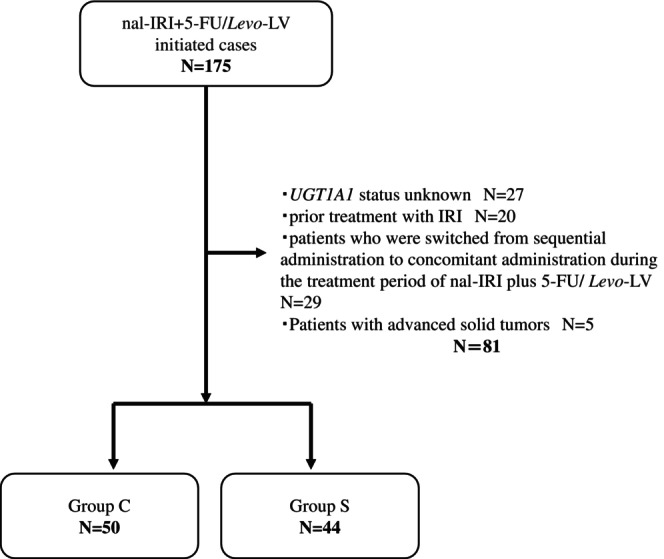
Flow diagram of patient selection. 5‐FU, 5‐fluorouracil; *Levo*‐LV, *Levo*‐leucovorin; Nal‐IRI, nanoliposomal irinotecan; UGT1A1, uridine‐diphosphate glucuronosyl transferase.

Electronic medical records of patients with locally advanced unresectable or metastatic pancreatic adenocarcinoma or adenosquamous carcinoma who received nal‐IRI plus 5‐FU/Levo‐LV therapy between June 1, 2020, and September 30, 2023, were retrospectively investigated. The selection criteria were age > 20 years, Eastern Cooperative Oncology Group performance status (ECOG PS) ≤ 2, refractory to or intolerant of a GEM‐based regimen, and adequate organ function. The exclusion criteria were as follows: *UGT1A1* status unknown, prior treatment with IRI, patients who were switched from sequential to concomitant administration during the treatment period of nal‐IRI plus 5‐FU/Levo‐LV, and patients with advanced solid tumors (Figure 2).

Our hospital has uniformly switched from sequential to concomitant administration at the end of September 2021. Patients were classified into a sequential administration group (Group S) or a concomitant administration group (Group C). In Group S, nal‐IRI (70 mg/m^2^ as IRI) was infused over 90 min, followed by *Levo*‐LV (200 mg/m^2^) over 120 min, and 5‐FU (2400 mg/m^2^) over 46 h. In Group C, nal‐IRI (70 mg/m^2^ as IRI) and *Levo*‐LV were infused simultaneously over 90 and 120 min, respectively, followed by 5‐FU 2400 mg/m^2^ intravenously over 46 h (Figure 1). The starting dose of irinotecan was 50 mg/m^2^ for patients homozygous for UGT1A1*6 or UGT1A1*28, or heterozygous for UGT1A1*6 and UGT1A1*28. When nal‐IRI was first introduced in our clinical practice, 5‐hydroxytryptamine 3 (5‐HT 3) receptor antagonist plus dexamethasone were administered as antiemetic prophylaxis. Neurokinin (NK)‐1 receptor antagonist was added at the discretion of the physician. With increasing experience, the combination of an NK‐1 receptor antagonist, 5‐HT 3 receptor antagonist and dexamethasone became routinely used as antiemetic prophylaxis. Adverse events (AEs) were assessed using the Common Terminology Criteria for Adverse Events (CTCAE) version 5.0. As an additional indicator of patient burden, the duration of hospital stay, the time from the start of the regimen to the transition to continuous 5‐FU infusion, was evaluated.

This study was conducted in compliance with the “Ethical Guidelines for Medical Research Involving Human Subjects” and approved by the Ethical Review Committee of Shizuoka Cancer Center (IRB number. J2022‐229‐2022‐1‐3). Information about the study inclusion criteria was posted in the hospital's bulletin, and consent was obtained via the opt‐out method.

### 2.2 Statistical Analyses

Fisher's exact test was used to compare the frequency of AEs between groups. Demographic and clinical factors included age, sex, ECOG PS performance status, pathology, *UGT1A1* status, metastatic organs, number of organ metastases, CA19‐9, modified Glasgow Prognostic Score (mGPS), neutrophil‐to‐lymphocyte ratio (NLR), treatment line, and antiemetic therapy. A cutoff value for NLR was defined as five, based on previous studies [6, 13]. The Mann–Whitney *U* test was applied to age and CA19‐9, whereas Fisher's exact test was used for the other parameters. The Mann–Whitney *U* test was used to compare the infusion times between the groups.

Log‐rank tests were performed to evaluate the PFS and OS. We confirmed the proportional hazard assumption of the Cox model. Analyses were conducted using data obtained up to the cut‐off date of November 30, 2023. OS was calculated as the time from the initiation of nal‐IRI to the date of death or confirmed survival, whereas PFS was calculated as the time from the start of nal‐IRI plus 5‐FU/*Levo*‐LV treatment to the date of death or disease progression. As a comparison of background factors of patients that affect prognosis, univariate Cox proportional hazards analyses were performed on patient background factors (Group, age, sex, ECOG PS, pathology, *UGT1A1* status, site of metastatic lesions, CA19‐9, mGPS, NLR, treatment line) for PFS and OS. Additionally, background factors with *p* < 0.1 found in the univariate analysis were selected for multivariate analysis. In this study, two cases had missing values for CA19‐9 and two for pathology. No imputation was performed for the missing data. All analyses were performed using EZR (version 4.2.2, Saitama Medical Center, Jichi Medical University, Saitama, Japan), a graphical user interface for R (The R Foundation for Statistical Computing, Vienna, Austria), with a two‐sided significance level of 5%.

## 3 Results

### 3.1 Patient Background

Among the 175 patients, 94 satisfied the inclusion criteria; 44 were classified in Group S and 50 in Group C (Figure 2). Table 1 shows patient characteristics by group. There were 31 patients with liver metastases in Group S (70.5%) and 17 in Group C (34.0%). Among patients with metastatic recurrence, 43 (97.7%) were in Group S and 37 (74.0%) in Group C. Patients were introduced after third‐line treatment, including 13 in Group S (29.5%) and 8 in Group C (16.0%).

**TABLE 1:** Baseline characteristics.

		Group S (*n* = 44)	Group C (*n* = 50)	*p* value
Age	Median (range), year	70 (57–80)	70 (50–89)	0.927
Gender	Male/Female	25/19	31/19	0.676
ECOG PS	< 2/2	17/27	19/31	1.000
Pathology	Adenocarcinoma/adenosquamous/UN	39/3/2	50/0/0	0.020
*UGT1A1* status	WT + SH/homo + double hetero	37/7	46/4	0.337
Site of metastatic lesions	Liver	31	17	< 0.001
	Peritoneum	12	17	0.510
	Lymph node	14	9	0.151
	Lung	11	6	0.116
Number of metastatic lesions	0/≥ 1	1/43	13/37	0.001
CA19‐9	Median (range), U/mL	665 (4–71 633)	426 (3–25 317)	0.677
mGPS	0/≥ 1	14/30	25/25	0.095
NLR	≥ 5.0/< 5.0	13/31	13/37	0.818
Treatment line	2nd/> 2nd	31/13	42/8	0.141
Antiemetic therapy	Aprepitant	34	42	0.442
	Dexamethasone	44	50	1.000
	Olanzapine	1	2	1.000
Initial dose reduction, yes		15	19	0.830
Dose reduction after 2nd course, yes	First	21	27	0.680
	Second	7	12	

*Note:* Mann–Whitney's U test was used for age and CA19‐9, and Fisher's exact test for the other items.

Abbreviations: CA19‐9, carbohydrateantigen19‐9; mGPS, modified Glasgow Prognostic Score; NLR, neutrophil‐to‐lymphocyte ratio; UN, unknown; WT + SH, wild type + single heterozygous.

### 3.2 Adverse Events

The incidence of grade 3 or higher AEs was comparable between the two groups, except for neutropenia, which showed a higher frequency in Group C than in Group S (9.1% vs. 16.0%; *p* = 0.368). Any grade of nausea (61.4% vs. 32.0%; *p* = 0.007), anorexia (65.9% vs. 42.0%; *p* = 0.024), and fatigue (56.8% vs. 30.0%; *p* = 0.012) was more frequent in Group S than in Group C; however, the incidence of grade 3 or higher AEs was low and did not differ between the two groups (Table 2). No febrile neutropenia or treatment‐related deaths were observed in either group.

**TABLE 2:** Adverse events.

	Group S (*n* = 44)		Group C (*n* = 50)		Group S vs. Group C *p* value	
	All grade	Grade 3/4	All grade	Grade 3/4	All grade	Grade 3/4
Neutropenia	21 (47.7%)	4 (9.1%)	24 (48.0%)	8 (16.0%)	1.000	0.368
Anemia	17 (38.6%)	4 (9.1%)	11 (22.0%)	1 (2.0%)	0.113	0.182
Thrombocytopenia	8 (18.2%)	1 (2.3%)	14 (28.0%)	2 (4.0%)	0.332	1.000
Nausea	27 (61.4%)	1 (2.3%)	16 (32.0%)	0 (0.0%)	0.007	0.468
Vomiting	4 (9.1%)	0 (0.0%)	1 (2.0%)	0 (0.0%)	0.182	1.000
Anorexia	29 (65.9%)	3 (6.8%)	21 (42.0%)	3 (6.0%)	0.024	1.000
Diarrhea	10 (22.7%)	0 (0.0%)	14 (28.0%)	0 (0.0%)	0.639	1.000
Fatigue	25 (56.8%)	4 (9.1%)	15 (30.0%)	1 (2.0%)	0.012	0.182
Stomatitis	4 (9.1%)	0 (0.0%)	2 (4.0%)	0 (0.0%)	0.414	1.000

*Note:* Numbers of patients are shown. *p‐*values are based on Fisher's exact tests.

### 3.3 Treatment Exposure

**FIGURE 3:**
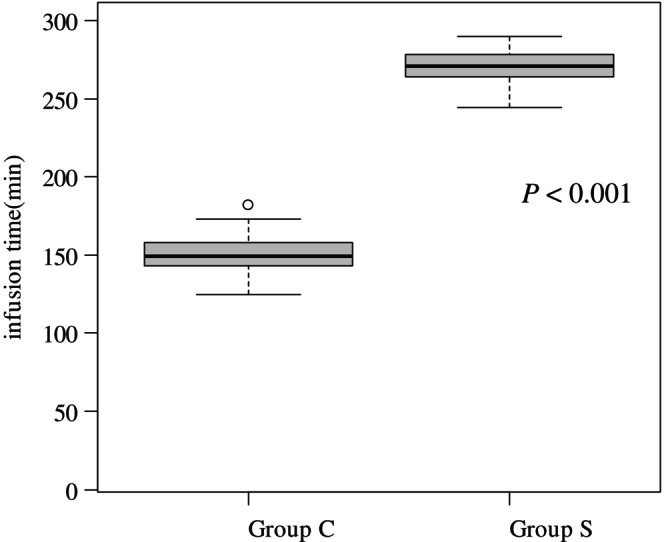
Duration of infusion in the hospital for each method of Group S on the right and Group C on the left.

Dose reductions during the first treatment cycle were observed in 15 (34.1%) patients in Group S and 19 (38.0%) in Group C (*p* = 0.830) (Table 1). The most common reasons for dose reduction were poor PS (13.6% vs. 18.0%), advanced age (13.4% vs. 17.1%), and AEs attributed to previous treatment (11.4% vs. 4.0%). Following the second cycle, dose reductions were required in 21 (47.7%) patients in Group S and 27 (54.0%) in Group C (*p* = 0.680) (Table 1). These were most frequently due to anorexia (47.6% vs. 25.9%), fatigue (28.6% vs. 33.3%), and nausea (38.1% vs. 14.8%). Subsequent anticancer therapy after study treatment discontinuation was delivered to 52.3% versus 54.0% (*p* = 1.000), with most patients receiving FOLFOX in both groups (Table 3). The median infusion time was significantly shorter in Group C than in Group S (271 min vs. 149 min; *p* < 0.001) (Figure 3).

**TABLE 3:** Treatment exposure.

	Group S (*n* = 44)	Group C (*n* = 50)	*p* value
On treatment	0 (0.0%)	5 (10.0%)	0.058
Median number of treatment courses (range)	4 (1–31)	7.5 (2–30)	0.015
Discontinuation	44 (100%)	45 (90.0%)	0.058
Refractory	42 (95.5%)	43 (86.0%)	
Intolerance^a^	2 (4.5%)	2 (4.0%)	
Post‐treatment	23 (52.3%)	27 (54.0%)	1.000
FOLFOX	21 (47.7%)	26 (52.0%)	
GEM + nab‐PTX	0 (0.0%)	1 (2.0%)	
S‐1	3 (6.8%)	3 (6.0%)	

*Note:* Mann–Whitney's U test was used for the median number of treatment courses, and Fisher's exact test for the other items.

Abbreviations: GEM + nab‐PTX, gemcitabine/nab‐paclitaxel; FOLFOX, 5‐fluorouracil/*levo*‐leucovorin plus oxaliplatin; S‐1, Tegafur/GiFmeracil/Oteracil.

^a^ Intolerance: PS reduction (*n* = 3), heart failure (*n* = 1).

### 3.4 Survival Time

**FIGURE 4:**
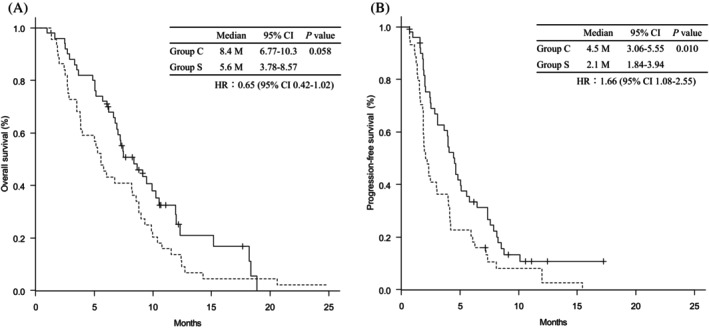
Kaplan–meier curve. (A) overall survival and (B) progression‐free survival in patients treated with nal‐IRI and 5‐FU/Levo‐LV. Comparison between Group C and Group S. Tick marks indicate censoring points. Solid lines indicate Group C, and broken lines indicate Group S. 5‐FU, 5‐fluorouracil; *Levo*‐LV, *Levo*‐leucovorin; Nal‐IRI, nanoliposomal irinotecan.

The median follow‐up time was 17.6 (range, 2.4–67.0) months for all randomized patients. Kaplan–Meier analysis showed that median PFS time was significantly shorter in Group S than in Group C (2.1 months [95% CI 1.84–3.94] vs. 4.5 months [95% CI 3.06–5.55]), with HR of 1.66 (95% CI, 1.08–2.55; *p* = 0.010 by log‐rank test). Median OS times were 5.6 months (95% CI 3.78–8.57) in Group S and 8.4 months (95% CI 6.77–10.3) in Group C, corresponding to an HR of 0.65 (95% CI 0.42–1.02; *p* = 0.058) (Figure 4). A univariate analysis was conducted to identify factors influencing PFS, and the background factors with *p* < 0.100 were administration method, pathology, presence of liver metastasis and CA19‐9. Covariates with a univariate *p* < 0.100 were entered into a multivariate Cox proportional hazards model, which identified Pathology (HR 3.52; 95% CI 1.01–12.2; *p* = 0.047) and CA19‐9 (HR 1.00; 95% CI 1.00–1.00; *p* = 0.007) as independent prognostic factors (Table 4A). On the other hand, univariate analysis was performed for OS, and the following background factors with *p* < 0.100 were identified: *UGT1A1* status and CA19‐9. These covariates were subsequently included in a multivariate Cox proportional hazards model. In the multivariate analysis, *UGT1A1* status (HR 0.46; 95% CI 0.22–0.97; *p* = 0.041) and CA19‐9 (HR 1.00; 95% CI 1.00–1.00; *p* = 0.001) were identified as independent prognostic factors for OS (Table 4B). In the multivariate analysis for PFS and OS, the method of administration was not identified as an independent prognostic factor.

**TABLE 4:** Univariate and multivariate analysis.

(A) For progression‐free survival							
Variables		Univariate analysis		*p* value	Multivariate analysis		*p* value
		HR	95% CI		HR	95% CI	
Group	S vs. C	0.59	0.38–0.90	0.014	0.64	0.40–1.04	0.070
Age	< 75/≥ 75	1.09	0.65–1.82	0.748			
Gender	Male/Female	1.04	0.67–1.61	0.872			
ECOG PS	< 2/2	0.55	0.13–2.24	0.403			
Pathology	Adenocarcinoma vs. adenosquamous	5.28	1.58–17.7	0.007	3.52	1.01–12.2	0.047
*UGT1A1* status	WT + SH/homo + double hetero	0.65	0.33–1.26	0.201			
Site of metastatic lesions	Liver	1.48	0.96–2.27	0.073	1.14	0.70–1.85	0.590
	Peritoneum	0.85	0.53–1.37	0.512			
	Lymphnode	1.22	0.74–1.99	0.440			
	Lung	1.13	0.65–1.98	0.668			
Number of metastatic lesions	0/≥ 1	1.65	0.90–3.05	0.108			
CA19‐9*	U/mL	1.00	1.00–1.00	0.002	1.00	1.00–1.00	0.007
mGPS	0/> 1	1.39	0.90–2.15	0.132			
NLR	≥ 5.0/< 5.0	1.26	0.78–2.03	0.349			
Treatment line	2nd/> 2nd	1.09	0.67–1.79	0.726			

(B) For overall survival							
Variables		Univariate analysis		*p* value	Multivariate analysis		*p* value
		HR	95% CI		HR	95% CI	
Group	S vs. C	0.69	0.45–1.08	0.102			
Age	< 75/≥ 75	1.32	0.80–2.17	0.280			
Gender	Male/Female	1.05	0.67–1.65	0.837			
ECOG PS	< 2/2	1.57	0.49–5.03	0.446			
Pathology	Adenocarcinoma vs. adenosquamous	1.46	0.46–4.66	0.521			
*UGT1A1* status	WT + SH/homo + double hetero	0.49	0.23–1.02	0.058	0.46	0.22–0.97	0.041
Site of metastatic lesions	Liver	1.44	0.93–2.24	0.103			
	Peritoneum	0.99	0.62–1.58	0.961			
	Lymphnode	1.07	0.65–1.76	0.797			
	Lung	1.20	0.68–2.12	0.525			
Number of metastatic lesions	0/≥ 1	1.80	0.87–3.74	0.116			
CA19‐9*	U/mL	1.00	1.00–1.00	0.001	1.00	1.00–1.00	0.001
mGPS	0/> 1	1.34	0.86–2.10	0.195			
NLR	≥ 5.0/< 5.0	1.18	0.72–1.92	0.518			
Treatment line	2nd/> 2nd	1.31	0.77–2.24	0.318			

*Note:* Univariate analysis was performed using Cox proportional hazards regression to evaluate the impact of patient background factors on PFS (4A) and OS (4B). *p‐*values, hazard ratios (HRs), and 95% confidence intervals for each factor were output. Multivariate analysis was performed for PFS (4A) and OS (4B) with *p‐*values less than or equal to 0.1 for each factor. All analyses were performed using EZR with a two‐sided significance level of 5%. CA19‐9* was analyzed as a continuous variable (per 1 U/mL).

Abbreviations: CA19‐9, carbohydrateantigen19‐9; mGPS, modified Glasgow prognostic score; NLR, neutrophil to lymphocyte ratio; WT + SH, wild type and single heterozygous.

## 4 Discussion

In this study, concomitant administration of nal‐IRI and *Levo*‐LV did not increase the incidence of grade 3 or higher AEs. Although OS with concomitant administration was comparable to that with sequential administration, PFS was significantly longer with sequential administration. These findings are consistent with those of the previous study [12]. Moreover, the infusion time was significantly shorter with concomitant administration, leading to reduced patient burden in hospital stay. In the multivariate analysis for PFS and OS, the method of administration was not identified as an independent prognostic factor. Concomitant administration of *Levo*‐LV with nal‐IRI may not increase adverse events or impact efficacy, while reducing infusion time.

When altering the administration methods, potential changes in the formulation owing to the concomitant administration of nal‐IRI and *Levo*‐LV were also considered. The diluent solutions for Nal‐IRI are defined as saline and 5% glucose solutions, with pH values of 4.5–8.0 [14] and 3.5–6.5 [15], respectively; nal‐IRI can theoretically be considered stable at 3.5–8.0 if we focus only on pH. *Levo*‐LV was shown to be stable from pH 1.36 to 12.60 in pH fluctuation tests [16]. The pH values of nal‐IRI and *Levo*‐LV solutions in clinical practice are 6.8–7.6 [5] and 6.02 [16] respectively, and the pH range is expected to be 6.02–7.6 when mixed in the infusion route. Therefore, the efficacy and safety of the solution administered via the intravenous drip route are unlikely to be affected, and the pH range is considered stable. In this study, the incidence of grade ≥ 3 AEs was similar in both the sequential and concomitant administration groups. Furthermore, no issues, such as catheter occlusion or crystallization, were observed during infusion, which is consistent with previous reports [12]. These findings suggest that there are no changes in the molecular structure of nal‐IRI or in the physicochemical properties of *Levo*‐LV.

There were differences in the frequency of AEs among administration methods. Grade 3 or higher neutropenia was more common in Group C. Primary prophylaxis with G‐CSF was not routinely implemented, and thus the impact of supportive care is considered minimal. Although the potential influence of the administration method cannot be excluded, this finding may be mainly explained by the trend toward a higher proportion of heavily pretreated patients in Group C. All grades of fatigue and nausea were more common in Group S. The higher proportion of aprepitant prescriptions in Group C compared with Group S (84.0% vs. 77.2%) suggests that improvements in supportive care may also have influenced these outcomes.

Median PFS was significantly longer in Group C than in Group S. Meanwhile, the method of administration was not identified as an independent prognostic factor in the multivariate analysis for PFS. Several factors may account for this finding. First, patients who switched from sequential to combination therapy were excluded; these patients have a more favorable prognosis, as they were able to continue nal‐IRI and 5‐fluorouracil combination therapy for a longer period. Second, a larger proportion of patients in Group S received treatment after the third line of therapy because they were awaiting approval of nal‐IRI. Conversely, although the proportion of patients with liver metastases (70.5% vs. 34.0%; *p* < 0.001) and those with metastatic lesions (97.7% vs. 74.0%; *p* = 0.001) was significantly higher in Group S, the multivariate analysis suggested that neither liver metastases nor metastatic lesions had a significant impact on survival in this study.

In this study, we used the intravenous infusion time in the hospital as an indicator of patient burden. Patients with advanced solid tumors spend 20%–25% of their survival time traveling to and from the hospital and undergoing treatment [17, 18, 19]. Frequent hospital visits place a burden on patients and caregivers in terms of time and psychological stress [18]. This time commitment can lead to unemployment or decreased income for individuals with limited flexibility in their work hours [20]. In general, treatment regimens requiring prolonged infusion times are increasingly viewed as problematic because of their negative impact on bed turnover rates. Treatments involving intravenous infusion tend to impose prolonged time constraints. Therefore, shortening infusion duration may contribute to alleviating the patients' time burden [21]. Since data regarding improvements in operational efficiency, such as the increase in the number of treatments administered per day, have not been collected, this remains an issue to be addressed in the future.

## 5 Limitations

This study had some limitations. First, it was a single‐center retrospective study with a small sample size, making selection bias an issue. Second, it was not possible to confirm whether the concomitant administration of nal‐IRI and *Levo*‐LV‐denatured liposomes would require blood samples. However, the safety profile data (particularly the pH data) indicated that the drug could be safely administered. Finally, given that our study was conducted at a single institution and involved only a Japanese population, further studies are warranted to assess the generalizability of the findings to other ethnic groups and institutions.

## 6 Conclusions

This study demonstrated that concomitant administration of nal‐IRI and Levo‐LV did not increase adverse events and showed comparable efficacy. Furthermore, it was associated with a shorter hospital stay, which may contribute to the reduction of time toxicity.

## Author Contributions

**Akane Wakabayashi:** data curation (lead), investigation (equal), writing – original draft (lead). **Rei Tanaka:** writing – review and editing (equal). **Yurie Fukumuro:** writing – review and editing (supporting). **Keita Mori:** formal analysis (lead), writing – review and editing (lead). **Junya Sato:** writing – review and editing (supporting). **Hiroshi Ishikawa:** writing – review and editing (supporting). **Michihiro Shino:** writing – review and editing (supporting). **Takeshi Kawakami:** conceptualization (lead), investigation (lead), methodology (lead), project administration (lead), supervision (lead), writing – review and editing (lead).

## Funding

The authors have nothing to report.

## Ethics Statement

This study was conducted in accordance with the Declaration of Helsinki and was approved by the Ethical Review Committee of Shizuoka Cancer Center (IRB number J2022‐229‐2022‐1‐3). Information about the study inclusion criteria was posted in the hospital's bulletin and consent was obtained via the opt‐out method.

## Conflicts of Interest

The authors declare no conflicts of interest.

## Data Availability

The data that support the findings of this study are available from the corresponding author upon reasonable request.
